# Plant growth acceleration using a transparent Eu^3+^-painted UV-to-red conversion film

**DOI:** 10.1038/s41598-022-21427-6

**Published:** 2022-10-26

**Authors:** Sunao Shoji, Hideyuki Saito, Yutaka Jitsuyama, Kotono Tomita, Qiang Haoyang, Yukiho Sakurai, Yuhei Okazaki, Kota Aikawa, Yuki Konishi, Kensei Sasaki, Koji Fushimi, Yuichi Kitagawa, Takashi Suzuki, Yasuchika Hasegawa

**Affiliations:** 1 Faculty of Engineering, Hokkaido University, Kita 13, Nishi 8, Kita-Ku, Sapporo, Hokkaido 060-8628 Japan; 2 Institute for Chemical Reaction Design and Discovery (WPI-ICReDD), Hokkaido University, Kita 21, Nishi 10, Kita-Ku, Sapporo, Hokkaido 001-0021 Japan; 3 Faculty of Agriculture, Hokkaido University, Kita 9, Nishi 9, Kita-Ku, Sapporo, Hokkaido 060-8589 Japan; 4 Graduate School of Agriculture, Hokkaido University, Kita 9, Nishi 9, Kita-Ku, Sapporo, Hokkaido 060-8589 Japan; 5 Graduate School of Chemical Sciences and Engineering, Hokkaido University, Kita 13, Nishi 8, Kita-Ku, Sapporo, Hokkaido 060-8626 Japan

**Keywords:** Organic molecules in materials science, Plant breeding, Light harvesting

## Abstract

The stimulation of photosynthesis is a strategy for achieving sustainable plant production. Red light is useful for plant growth because it is absorbed by chlorophyll pigments, which initiate natural photosynthetic processes. Ultraviolet (UV)-to-red wavelength-converting materials are promising candidates for eco-friendly plant cultures that do not require electric power. In this study, transparent films equipped with a UV-to-red wavelength-converting luminophore, the Eu^3+^ complex, were prepared on commercially available plastic films for plant growth experiments. The present Eu^3+^-based films absorb UV light and exhibit strong red luminescence under sunlight. Eu^3+^-painted films provide significant growth acceleration with size increment and biomass production for vegetal crops and trees in a northern region. The plants cultured with Eu^3+^-painted films had a 1.2-fold height and 1.4-fold total body biomass than those cultures without the Eu^3+^ luminophores. The present film can promote the plant production in fields of agriculture and forestry.

## Introduction

Sustainable scientific and technological developments for future plant production are among the most important challenges associated with supplying sufficient global food and bioenergy in terms of world population growth, which is expected to reach nearly nine billion people by 2050^1–4^. Stimulating photosynthesis is a promising strategy to accelerate plant growth and biomass production^5,6^. Light energy is required by natural photosynthetic organisms such as higher plants, algae, and photosynthetic bacteria. In the initial stage of photosynthesis, chlorophyll pigments absorb sunlight and utilize solar photon energy for the production of bio-resources^7^. In particular, land plants contain the chlorophyll-*a* and *b* pigments in light-harvesting antenna and reaction center apparatuses, which absorb visible light in the red (Qy band: 600–700 nm) and blue (Soret band: 400–450 nm) regions. The effective wavelengths for photosynthesis agree well with the absorption bands. The light color also affects plant growth, productivity, morphology, and physiology^8–10^. The photosynthetic action spectra have revealed that red light for excitation in the Qy band provides the highest photosynthetic quantum yield^11^.

**Figure 1:**
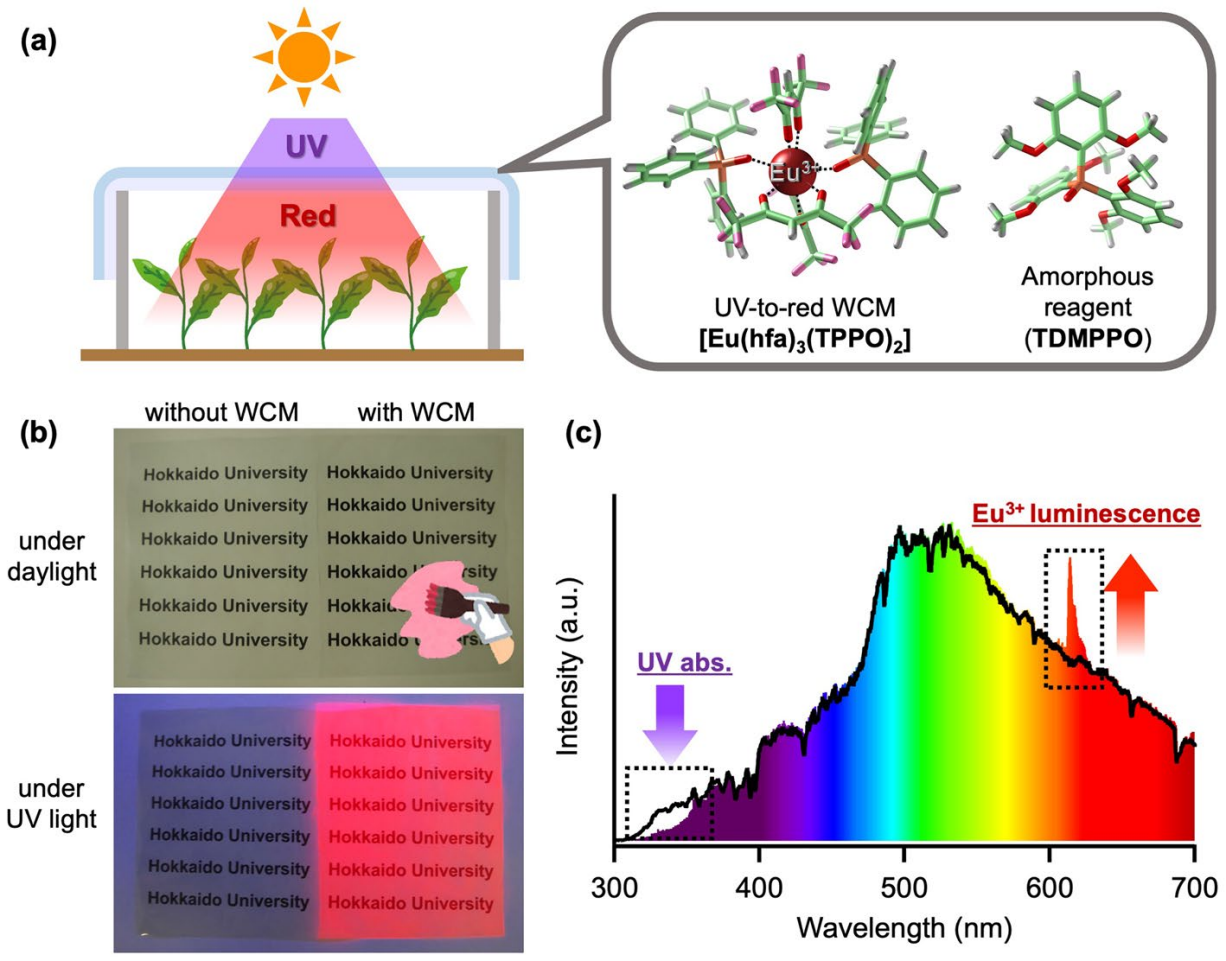
Transparent UV-to-red WCM film equipped with a Eu^3+^ luminophore. (**a**) Schematic of plant growth acceleration using WCM film (left) and molecular structures of [**Eu(hfa)**_**3**_**(TPPO)**_**2**_] and **TDMPPO** (right). (**b**) Photographs of agriculture film painted with or without WCM under daylight (upper) and UV irradiation (lower). (**c**) Solar spectra obtained without (black line) and with the WCM film (solid colors).

Sunlight is a sustainable energy source, however, it includes ultraviolet (UV) light, which is located in the high-energy wavelength region (200–400 nm). Various plants respond to UV light^12–14^. In particular, UV-A (320–400 nm) light affects not only photo-inhibition of photosystem(PS)-II^15^ but also increase of photosynthetic rate^16^. UV-B (295–320 nm) is known to induce damage of DNA^15^. Thus, shading the plants from solar UV light is one of the key strategies to suppress photoinhibition and photodamages of the plants. The plastic covering films used in greenhouses contain UV-blocking materials. The UV-blocking molecules convert UV light into thermal energy. In the past decade, wavelength-converting materials (WCM) have been reported to change the UV to red light for plant cultivation^17–21^. Utilizing WCMs under sunlight can be advantageous for filtering solar UV light and providing red light for efficient photosynthesis. The WCMs can promote sustainable agriculture and forestry without consuming the electricity such as plant factories equipped with LED devices. The trivalent europium (Eu^3+^) luminophore is a promising candidate for sustainable UV-to-red WCM^22–24^. The Eu^3+^ complex exhibits strong red luminescence based on the 4*f*–4*f* transitions by the attachment of organic ligands. The Eu^3+^ luminophore with a photosensitizer (hexafluoroacetylacetonato: hfa) and a stabilizer (triphenylphosphine oxide: TPPO), [**Eu(hfa)**_**3**_**(TPPO)**_**2**_] (Fig. 1a and Supplementary Fig. S1), shows a high conversion efficiency from UV to red light (emission quantum yield ≃ 70%) with almost no absorption bands in the visible-light region^25^. This material is also stable upon heating at 200 °C^26^ and under UV light irradiation^27^. Recently, we reported that a Eu^3+^ complex with tris(2,6-dimethoxyphenyl)phosphine oxide (**TDMPPO**, Fig. 1a and Supplementary Fig. S1) produces a transparent amorphous paint with red luminescence under UV light exposure^28^.

In this study, we first demonstrated plant growth acceleration using a novel transparent UV-to-red WCM with a strongly luminescent Eu^3+^ complex for agriculture and forestry under sunlight of the greenhouse conditions (Fig. 1a). The transparent UV-to-red WCM-film was prepared by coating a mixture of luminophore [**Eu(hfa)**_**3**_**(TPPO)**_**2**_] and the amorphous reagent **TDMPPO** on a commercially available plastic covering film for plant growth experiments. The Eu^3+^-painted WCM films significantly promoted the growth in size and biomass production of vegetal and tree plants.

## Results and discussion

### Preparation of a transparent Eu^3+^-painted WCM film

A strongly luminescent Eu^3+^ complex, [**Eu(hfa)**_**3**_**(TPPO)**_**2**_], was used as a UV-to-red WCM. The amorphous formation of [**Eu(hfa)**_**3**_**(TPPO)**_**2**_] was analyzed using powder X-ray diffraction (PXRD). The PXRD patterns of [**Eu(hfa)**_**3**_**(TPPO)**_**2**_] and **TDMPPO** showed specific peaks due to the crystallinity of the compounds (Supplementary Fig. S2). [**Eu(hfa)**_**3**_**(TPPO)**_**2**_] and **TDMPPO** in a 1:2 molar ratio were dissolved in dichloromethane and evaporated to give a mixed solid. The obtained mixture exhibited broad PXRD signals, indicating that the mixture formed an amorphous phase (Supplementary Fig. S2).

A mixture of [**Eu(hfa)**_**3**_**(TPPO)**_**2**_] and **TDMPPO** was painted on a commercially available polyolefin-type covering film (thickness: 0.1 mm, C. I. Takiron Co., Osaka, Japan). The painted film was appeared to have clear transparency and bright red luminescence under UV irradiation (Fig. 1b). Confocal laser scanning microscopic analysis showed that the thickness of the painted film was approximately 60 µm (Supplementary Fig. S3). The amorphous Eu^3+^-based WCM on the polyolefin-type film was exposed to sunlight. The WCM film exhibits characteristic Eu^3+^ luminescence at 614 nm and absorption in the UV region (Fig. 1c). The intensity of solar light in the visible region was not decreased. These results revealed that the film covered with an amorphous Eu^3+^ complex performed as UV-to-red WCM without daylight suppression. In addition, WCM film had little influence on temperature of plant surface under sunlight (Supplementary Fig. S9).

The photophysical properties of the painted UV-to-red WCM films were evaluated in detail using spectroscopic techniques. The UV–Vis absorption spectra of the WCM film showed a π–π* transition band at 298 nm, which was attributed to the hfa ligands (Supplementary Fig. S5). The luminescence spectrum of the WCM film under UV irradiation exhibited characteristic emission peaks of the Eu^3+^ complex at 578, 592, 614, 650, and 699 nm, which correspond to the 4*f*–4*f* transitions derived from ^5^D_0_ → ^7^F_*J*_ (*J* = 0, 1, 2, 3, and 4), respectively (Supplementary Fig. S6). The excitation spectrum of the WCM film detected at the 614 nm emission also showed a π–π* transition band at 298 nm, which was similar to the UV–Vis absorption spectrum of WCM film (Supplementary Fig. S7). The luminescence lifetime of the amorphous Eu^3+^ complex was estimated to be 0.81 ms (Supplementary Fig. S8). Based on these photophysical data, the quantum efficiency of the 4*f*–4*f* transitions was calculated to be 64% (Supplementary Table S1). The radiative and non-radiative constants of the amorphous Eu^3+^ complex were calculated to be 7.9 × 10^2^ and 4.4 × 10^2^ s^−1^, respectively (Supplementary Table S1). The photophysical performance of the Eu^3+^-painted WCM film was similar to that of the original [**Eu(hfa)**_**3**_**(TPPO)**_**2**_] powder. A bright luminescent Eu^3+^-painted UV-to-red WCM film was successfully prepared for the plant growth experiments. The photophysical properties of the WCM film after the plant culture experiments were also analyzed by above spectroscopic methods (Supplementary Figs. S4, S6 and S8 and Supplementary Tables S1 and S2). The employed WCM films still exhibited red luminescence under UV light irradiation (Supplementary Fig. S4).

### Hydroponic culture and growth of vegetal crops

**Figure 2:**
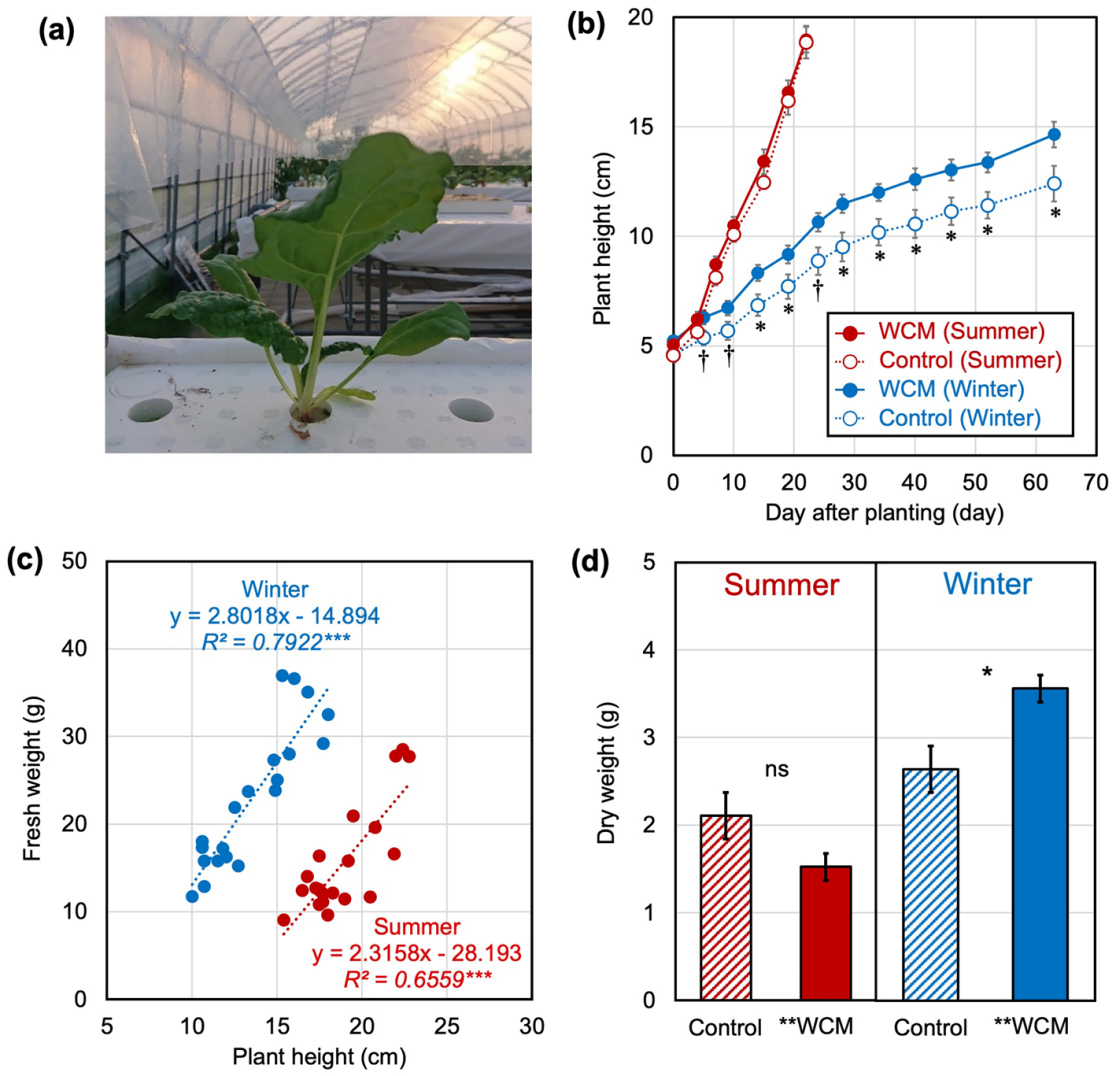
Culture and growth analysis of Swiss chard. (**a**) Photograph of hydroponic culture of Swiss chard covered with WCM film. (**b**) Plant heights of hydroponic cultured Swiss chard. (**c**) Relationships between the plant height and the leaf yield calculated from the data at the harvest time in summer and winter. (**d**) Total plant biomass of hydroponic cultured Swiss chard. The plant height represents the average ± standard error (*n* = 10). The symbols * and † represent significance at 5% and 10% of Student’s t-test.

The WCM film was used for plant growth experiments on vegetal crops under sunlight (Fig. 2a). Ten Swiss chard plantlets were hydroponically cultured with and without WCM films in summer and winter. In summer, the Swiss chard cultured reached the plant size required for the harvest in 22 days, however, those cultured in winter took 60 days to reach the size for harvesting (Fig. 2b). The difference in harvest age originates from the ambient temperature during the season. The plant height at harvest can be monitored by non-destructive inspection, showing a close correlation with the yield during summer and winter independently (Fig. 2c). In winter, the plant height of Swiss chard cultured with the WCM film was 1.2-fold higher than that without the film. The effect of the WCM film was maintained during the experimental period (63 days). The total body biomass increased for the harvested Swiss chard cultured with the WCM film in winter, which was 1.4-fold larger than that without the WCM film (Fig. 2d). In summer, the plant height of Swiss chard cultured with the WCM film was similar to that without the WCM film. The specific effect of WCM films on total body biomass was also not observed in summer. These results indicate that the WCM film specifically promotes plant growth in winter. Although plant growth is normally suppressed in winter, the WCM film accelerates plant growth even under weak solar radiation intensity, short daytime, and low ambient temperature. The WCM film promoted the yield of leafy vegetables to a marketable size in comparison to previous reports^23,24^. We consider that the effective acceleration of plant growth by the WCM film is due to the accumulation of red light during long-term plant cultivation.

### Culture and growth of tree plants

**Figure 3:**
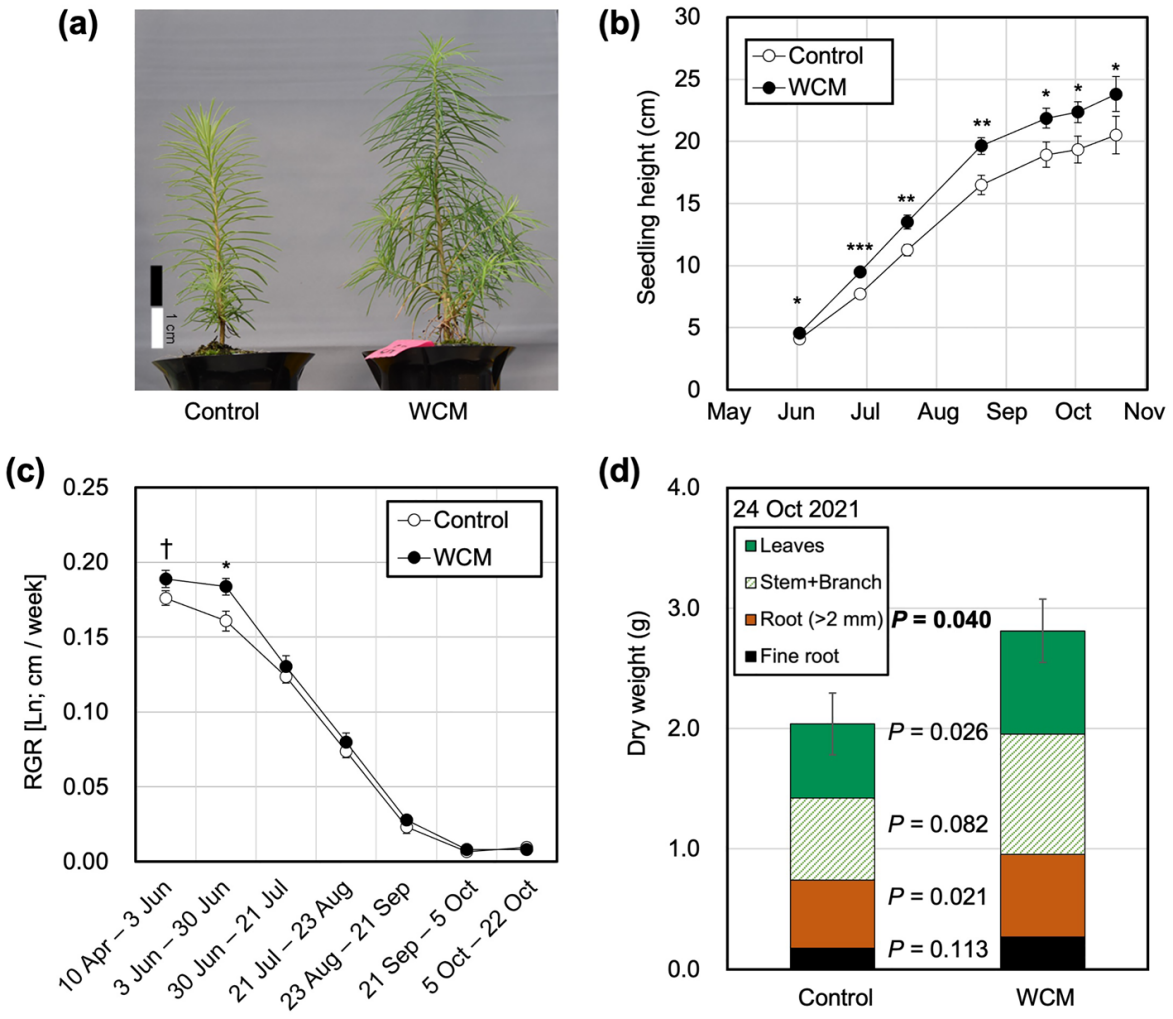
Growth of Japanese larch tree seedlings under UV-to-red WCM film. (**a**) Photograph of 4-month-old Japanese larch seedling grown under UV-to-red WCM film and non-treated film (control). (**b**) Seasonal change of seedling height. (**c**) Seasonal change of RGR. RGR was normalized by natural logarithm per week. Open and closed circles denote treatments of the control and WCM films, respectively. Error bars denote standard error. Statistical significance was examined in each month by Student’s t-test (*n* = 81 and 70, ^†^*P* < 0.1, ^*^P < 0.05, ^**^P < 0.01, ^***^P < 0.001). (**d**) Dry weight of fine roots, thick roots, stem and branch, and leaves. Error bars denote standard error of total biomass. Statistical significance was examined for each organ and whole body by Student’s t-test (*n* = 38 and 36).

Japanese larch trees were cultured using WCM films under sunlight. During one growing season for Japanese larch tree seedlings, the performance of the WCM film as an accelerator to initial growth of a perennial plant was evaluated in a culture experiment (Fig. 3a). We observed significantly higher heights of larch tree seedlings 3 months after sowing (3rd, June 2021). The size of the seedlings with the WCM films was larger than that without the films (control) until the end of the growing season, 22, October 2021 (Fig. 3b). The relative growth rate (RGR) in culture with the WCM film until the end of June was significantly higher than that without WCM. We did not observe a drastic difference between the RGR with and without WCM films in summer and autumn (Fig. 3c). These results indicate that the WCM films positively affected the height growth and acceleration of the initial growth of Japanese larch trees. At the end of the cultivation experiment, the diameter of the stem at soil surface level was 1.2-fold larger in seedlings under WCM film than those under the untreated films (3.70 ± 0.20 vs. 4.51 ± 0.21 mm [± SE]; Student’s t-test, *P* < 0.001). The total biomass of seedlings was 1.4-fold greater in seedlings under WCM film, with significant increments in thick roots, branches, stems, and leaves (Fig. 3d). A drastic increase in size and biomass was clearly observed using the WCM films. By cultivation with WCM films, the seedling size of a one-year-old larch tree could reach the standard rank for available planting in the forestry of Hokkaido, Japan (> 25 cm seedling height and > 4 mm diameter). The WCM films can shorten the growth period of seedlings from two to one year, contributing to cost-effective production.

## Conclusion

A novel UV-to-red WCM film was prepared by the amorphous formation of a strongly luminescent Eu^3+^ complex. The present WCM film accelerates the increase in size and biomass of vegetal crops and trees under sunlight. WCM can be easily painted on a commercially available plastic film, and its UV-to-red conversion is achieved by sunlight irradiation without any electric power. Thus, the WCM film can shorten the period of plant production and provide cost-effective and eco-friendly plant growth promotion for agriculture, horticulture, and forestry. It is also important to study the effective plant species for growth acceleration and biomass production using WCM films in future. This painting technology using WCM can be useful for applications in glass-type greenhouses. The **Eu(hfa)**_**3**_**(TPPO)**_**2**_ luminophore has been commercially available, and outside-painted WCM films were not directly attached with plants. In our experiments, the thickness of painted film was found to be 60 μm. Absorption coefficient of the Eu^3+^ complex at UV area is estimated to be approximately 30,000 cm^−1^ M^−1^ at 310 nm. Paint-thickness of this WCM is sufficient for effective absorption of UV light. Overcoating process of WCM may be effective for plant growth. Future luminophore studies and painting technologies promise to provide UV-to-red WCM films with high durability, longevity, high quantum yield, low cost, and appropriate thickness for the plant culture. We also believe that UV-to-red WCM technology can contribute to sustainable development goals (SDGs): SDG2 (zero hunger), SDG7 (affordable and clean energy), SDG9 (industry, innovation, and infrastructure), and SDG15 (life on land).

## Materials and methods

### Preparation of UV-to-red WCM film

UV-to-red WCM, [**Eu(hfa)**_**3**_**(TPPO)**_**2**_] (Lumisis E-300, CentralTechno Co., Osaka, Japan), was used without further purification. The amorphous reagent, **TDMPPO**, was prepared according to a reported procedure^28^ from commercially available tris(2,6-dimethoxyphenyl)phosphine (Tokyo Chemical Industry Co., Ltd., Tokyo, Japan). The solvents were purchased from Kanto Chemical Co. and used without further purification. A polyolefin-type film (Coating 5 + 1, thickness: 0.1 mm, C. I. Takiron Co., Osaka, Japan) was used for the agricultural plastic covering film. The UV-to-red WCM films were prepared by painting a dichloromethane solution consisting of [**Eu(hfa)**_**3**_**(TPPO)**_**2**_] (20 mM) and **TDMPPO** (40 mM) on polyolefin-type plastic films.

### Measurements of WCM film

UV–Vis absorption spectrum was measured in transmission mode by a JASCO V-670 spectrophotometer. The luminescence and excitation spectra were recorded on a HORIBA Fluorolog-3 spectrofluorometer and corrected for the response of the detector system. The luminescence lifetimes were measured using the third harmonics (355 nm) of a Q-switched Nd:YAG laser (Spectra Physics, INDI-50, FWHM = 5 ns, λ = 1064 nm) and a photomultiplier (Hamamatsu Photonics, R5108, response time ≤ 1.1 ns). The Nd:YAG laser response was monitored with a digital oscilloscope (Sony Tektronix, TDS3052, 500 MHz) synchronized to the single-pulse excitation. The emission quantum yields (excitation wavelength: 330 nm) for the WCM films were measured using a JASCO F-6300-H spectrometer attached to a JASCO ILF-533 integrating sphere unit (Φ = 100 mm) in air. The solar radiation spectra were obtained using an Ocean Photonics USB4000 spectrophotometer. The PXRD data were recorded on a Rigaku SmartLab diffractometer with Cu-Kα radiation and a D/teX Ultra detector. Confocal scanning laser microscopy was performed using a Keyence VK-8710 with a 658 nm laser. Temperature of plant surface was measured by infrared camera (NS9500, InfReC, Nippon Avionics Co. Ltd., Kanagawa, Japan).

### Plant materials

The ‘Bietola Bianco’ cultivar of Swiss chard (*Beta vulgaris* var. *cicla* (L.) K.Koch) was used as the experimental material. The high-functioning cultivar has green leaves and white stalks, which is suitable for the hydroponic culture systems.

The Japanese larch (*Larix kaempferi* (Lamb.) Carr.) was used as the experimental material. This species is light-demanding and grows relatively fast compared to other conifer trees. Larch species are a major forestry trees in the northern biosphere.

### Place for plant culture experiments

The cultivation experiment was conducted in a greenhouse at the Research and Education Center for Robust Agriculture, Forestry and Fisheries Industry, Hokkaido University, Sapporo, northern Japan (43°04′N, 141°20′E, 15 m a.s.l.).

### Hydroponic culture of Swiss chard

Before seeding, all seeds (TOKITA SEED Co., Ltd., Saitama, Japan) were sterilized with 70% ethanol for 30 s and rinsed with sterilized distilled water. The seeds were germinated in the dark on wet filter paper in an incubator at 25 °C for several days. After 80% seed germination, the seedlings were transferred to polyurethane foams and then planted in a hydroponic culture system using the nutrient film technique (NFT) in a greenhouse. The root was immersed in a hydroponic solution (guaranteed soluble compounds: N, 80 mg L^−1^; P, 76 mg L^−1^; K, 188 mg L^−1^; Mg, 20 mg L^−1^; Mn, 0.54 mg L^−1^; B, 1.1 mg L^−1^, 500 times diluted solution of Hyponica (Kyowa Co., Ltd., Osaka, Japan). The pH and electric conductivity (EC) were adjusted to 6.5–6.8 and 1.8–2.4 mS cm^−1^, respectively. The temperature of the hydroponic solution was controlled to 18–22 °C. The hydroponic culture was performed twice: the summer experiment from September 2018 to October 2018, and the winter experiment from October 2018 to January 2019.

To evaluate the responses of the plants to WCM, a simple environment covered with WCM film was created, except for the control treatment without film covering (control). Ten plantlets were cultivated for each treatment and harvested when their plant height reached 20 cm.

### Analysis of Swiss chard

Plant height was represented as the maximum length calculated from the sum of the leaf blade and petiole and was measured every 1–2 weeks. The total body biomass above the ground of the Swiss chard was weighed at harvest. The data for each replicate were calculated as the average value for the plantlets. The experiment was arranged in a randomized block design. Statistical analyses, including Student’s t-test and Pearson’s correlation coefficients, were conducted using Statcel4 (OMS, Tokyo, Japan), an add-in form in Microsoft Excel 2004 for Mac.

### Cultural condition of larch tree seedlings

To evaluate plant growth in response to WCM, two types of plots covered with WCM film and non-WCM film (control) were set up in the greenhouse. The WCM and control plots were repeated three times to form six plots on a line. Before seeding of Japanese larch, all seeds collected in Yubetsu Town, Hokkaido, Japan (mother tree ID: S199) were well-washed and subjected to a cold stratification at 4 °C for one month. The plastic pots (330 mL with inside ribs and slits, HSK330, Hokkaido forests seeds and seedling cooperative association) were filled with a commercial-blend soil medium (coco-peat : Kanuma-soil = 4: 1 (vol/vol); pH 6.0, EC = 0.2 mS cm^−1^, N = 500 mg L^−1^, P = 900 mg L^−1^, K = 750 mg L^−1^; Container seedling raising soil, Top Co., Ltd, Osaka, Japan), adequately wetted by tapped water. At the end of February, the seeds were sown on potted soils one by one with a cover of a thin soil layer. The automatic spray mist by tap water was irrigated twice a day in the early morning and evening for 5 min in spring and autumn or 10 min in summer. In April, 160 seedlings were grown. In each plot, the positions of the seedling pots were fully randomized and rotated. Additional fertilization of 1000 times diluted solution of HYPONeX (HYPONeX Japan Co., Ltd., Osaka, Japan) was treated 10 times on a weekly basis from 2nd June to August 2021.

### Data collection and analysis of Japanese larch seedlings

The seedling height was measured monthly from June to October 2021. The stem diameter was measured at the soil surface level using a caliper with 0.01 mm accuracy. Half of the seedlings were screened based on their size to reappear in the data distribution and harvested at the end of the growing season, 24, October 2021. After harvest, leaves, twigs, stems, thick roots (> 2 mm in diameter), and fine roots (less than 2 mm) were respectively dried at 70 °C for over 3 days, and the dry weight was measured. The RGR of seedling height was calculated as the ratio of height at the end to the initial height of each period normalized by natural logarithm per week. Statistical analysis was performed using Student’s t-test at every time point.

### Guideline statement

The authors confirm that all procedures of the experiments, including the collection of plant materials and culturing the plants, complied with relevant institutional, national, and international guidelines and legislation.

## Supplementary Information

Supplementary Information.

## Data Availability

The data that support the findings of this study are available from the corresponding authors on reasonable request.
