# Supplementary material for: Plant growth acceleration using a transparent Eu3+-painted UV-to-red conversion film
Source: Sci Rep. 2022 Oct 26;12:17155. doi: 10.1038/s41598-022-21427-6 (PMC9605945; doi:10.1038/s41598-022-21427-6)
Supplement: Supplementary file 1 — Supplementary Information. [file 41598_2022_21427_MOESM1_ESM.docx]

**Supplementary Information**

**Plant growth acceleration using a transparent Eu^3+^-painted UV-to-red conversion film**

Sunao Shoji,^1,2,*^ Hideyuki Saito,^3,*^ Yutaka Jitsuyama,^3^ Kotono Tomita,^4^ Qiang Haoyang,^4^ Yukiho Sakurai,^4^ Yuhei Okazaki,^4^ Kota Aikawa,^5^ Yuki Konishi,^5^ Kensei Sasaki,^5^ Koji Fushimi,^1^ Yuichi Kitagawa,^1^ Takashi Suzuki,^3,*^ Yasuchika Hasegawa^1,2,*^

^1^Faculty of Engineering, Hokkaido University, Kita 13, Nishi 8, Kita-ku, Sapporo, Hokkaido 060-8628, Japan.

^2^Institute for Chemical Reaction Design and Discovery (WPI-ICReDD), Hokkaido University, Kita 21, Nishi 10, Kita-ku, Sapporo, Hokkaido 001-0021, Japan.

^3^Faculty of Agriculture, Hokkaido University, Kita 9, Nishi 9, Kita-ku, Sapporo, Hokkaido 060-8589, Japan.

^4^Graduate School of Agriculture, Hokkaido University, Kita 9, Nishi 9, Kita-ku, Sapporo, Hokkaido 060-8589, Japan.

^5^Graduate School of Chemical Sciences and Engineering, Hokkaido University, Kita 13, Nishi 8, Kita-ku, Sapporo, Hokkaido 060-8628, Japan.


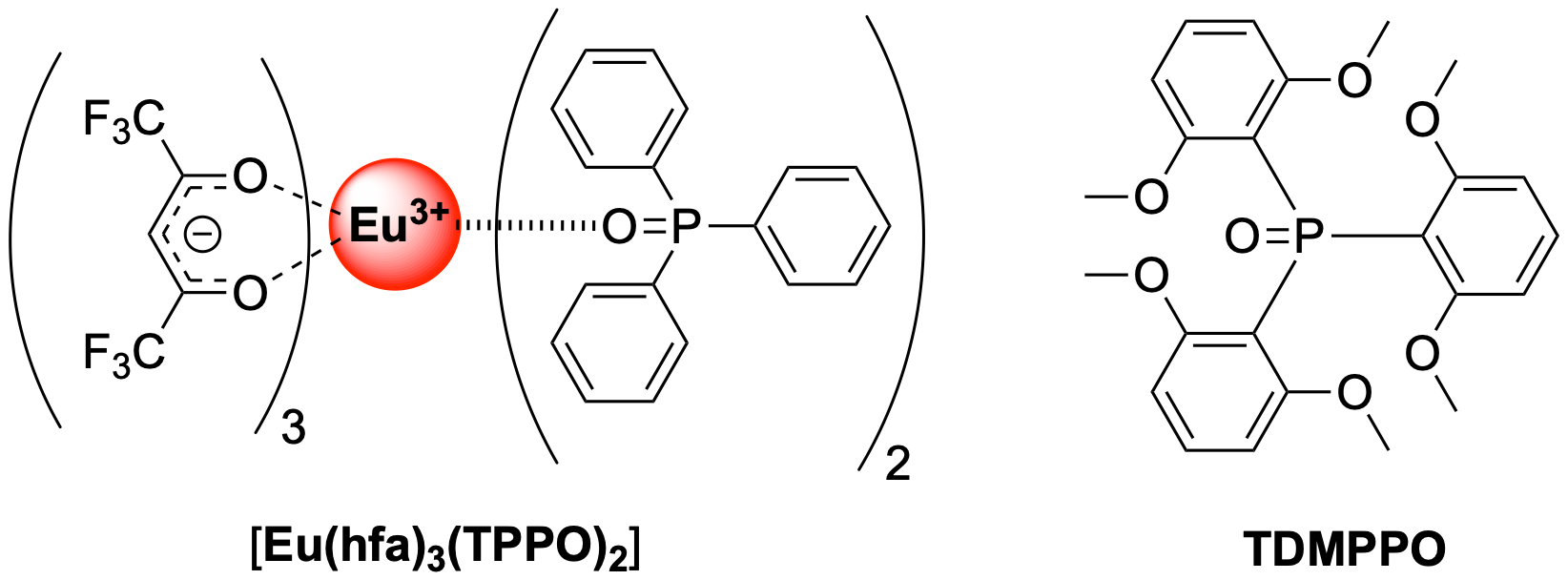


**Supplementary Figure S1.** Molecular structures of [**Eu(hfa)_3_(TPPO)_2_**] (left) and **TDMPPO** (right).


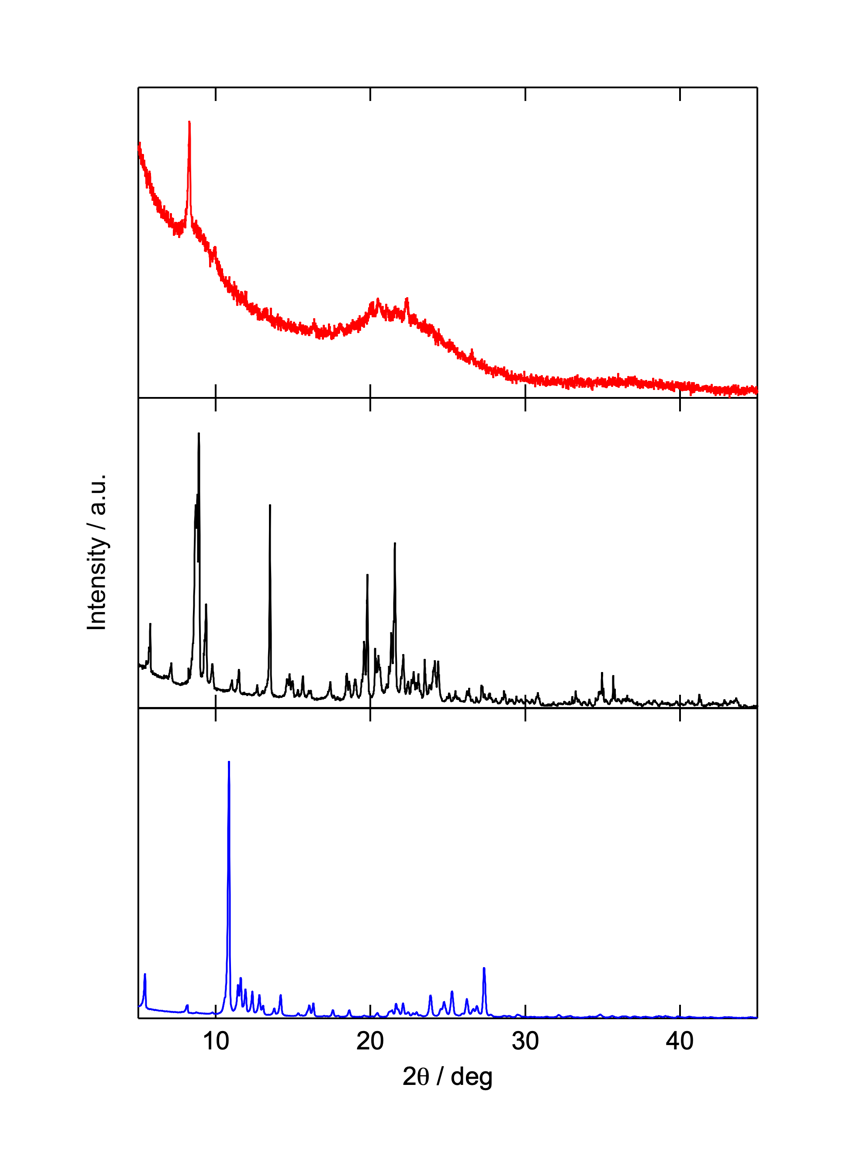


**Supplementary Figure S2.** PXRD profiles of WCM (**Eu(hfa)_3_(TPPO)_2_**/**TDMPPO** = 1/2 (mol/mol)) (upper, red line), **Eu(hfa)_3_(TPPO)_2_** (middle, black line), and **TDMPPO** (lower, blue line).

**
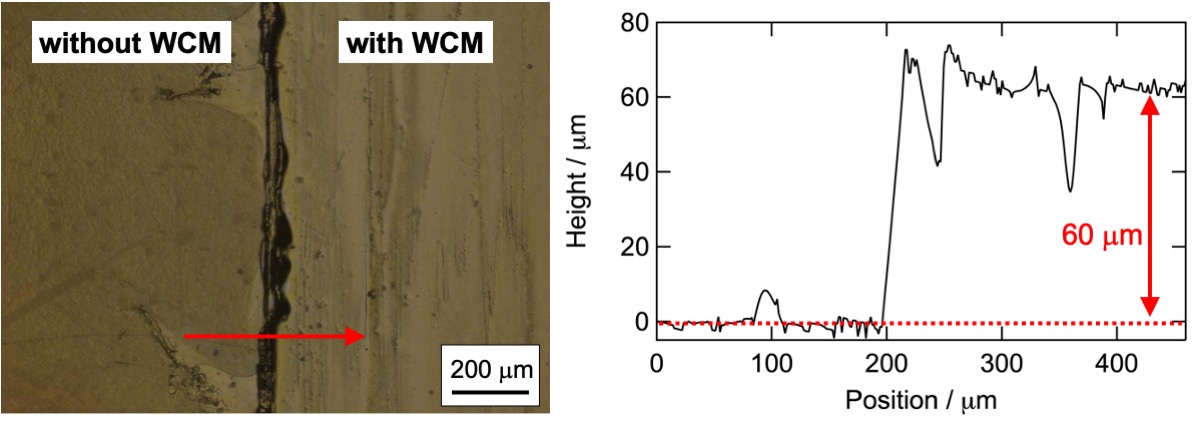
**

**Supplementary Figure S3.** Microscopic analyses of a plastic covering film coated with a Eu^3+^-based WCM. Confocal laser scanning microscopic image (left) and cross-section analysis along a red arrow in a microscopic image (right).

**
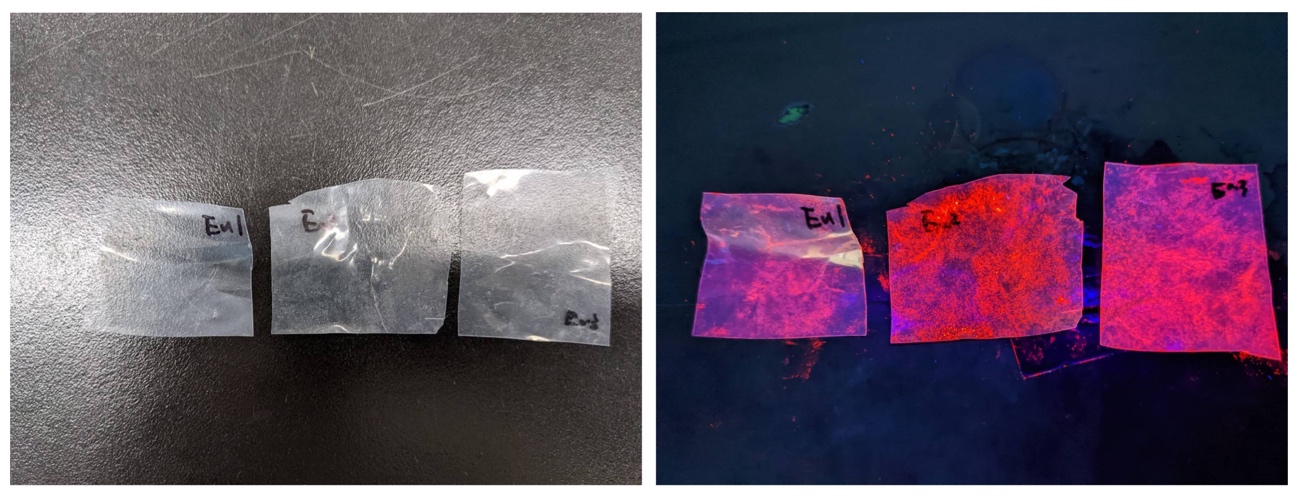
**

**Supplementary Figure S4.** Photographs of Eu^3+^-based WCM films after plant growth experiments under daylight (left) and UV light (right).

**
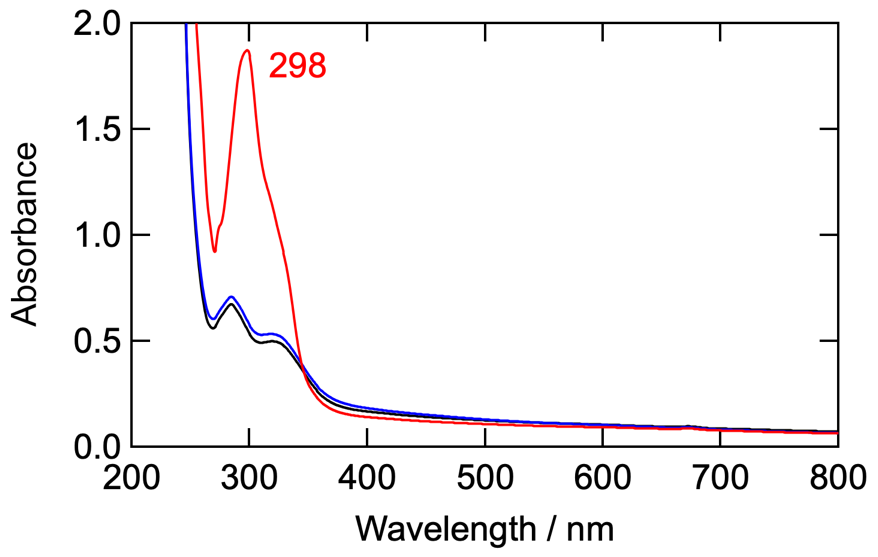
**

**Supplementary Figure S5.** UV-Vis absorption spectra of plastic covering film (black), film with painting only dichloromethane (blue), and film with painting a dichloromethane solution of Eu^3+^-based WCM (red).

**
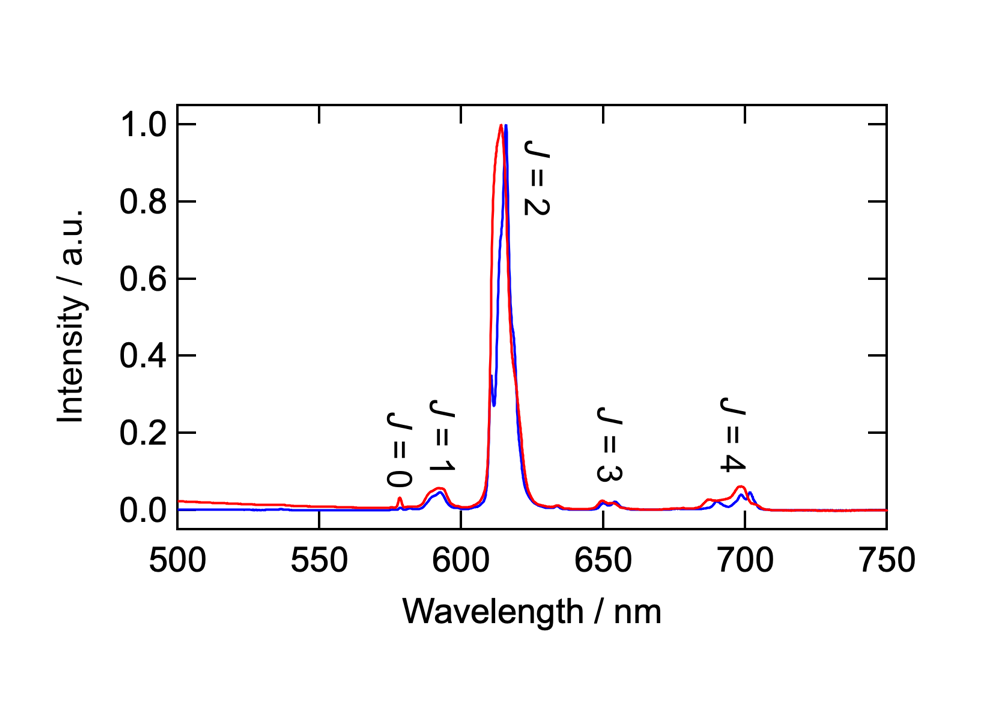
**

**Supplementary Figure S6.** Luminescence spectra of a Eu^3+^-based WCM film on a plastic covering film (λ_ex_ = 360 nm) before (red line) and after plant growth experiment (blue line).

**
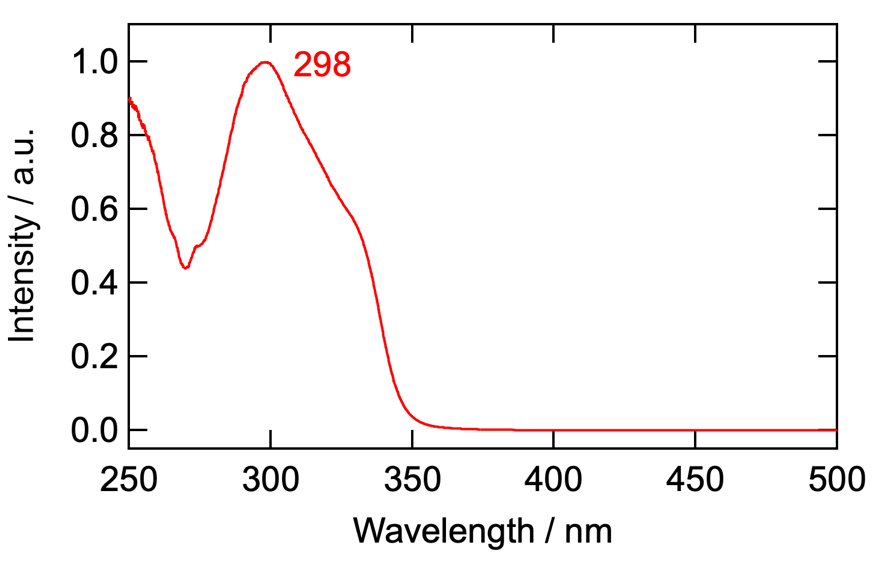
**

**Supplementary Figure S7.** Excitation spectrum of a Eu^3+^-based WCM film on a plastic covering film (λ_em_ = 613 nm).

**
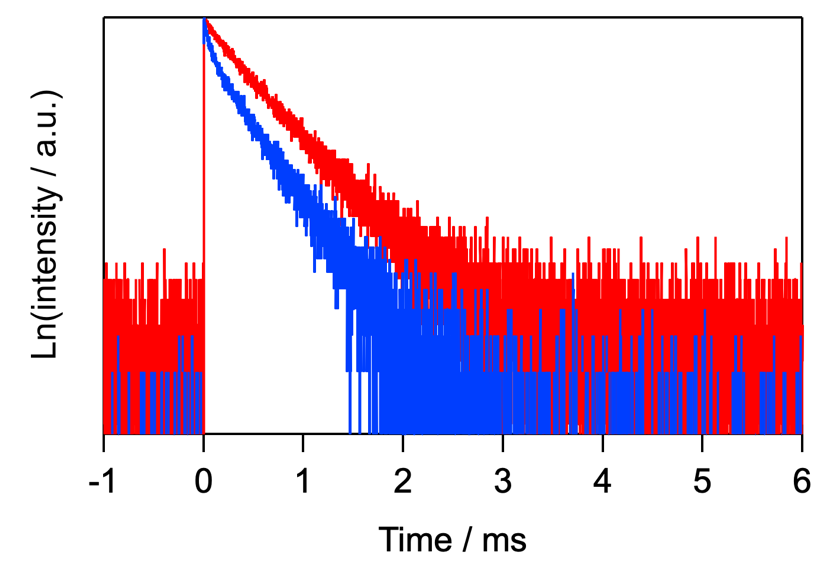
**

**Supplementary Figure S8.** Luminescence decay profiles (λ_ex_ = 355 nm) of a Eu^3+^-based WCM film before (red line) and after plant growth experiment (blue line).

The emission quantum yield excited by 4*f*-4*f* transition (Φ*_f-f_*), the radiative (*k*_r_) and nonradiative (*k*_nr_) rate constants were estimated using the following equations (1)–(3):

$\text{k}_{\text{r}\text{ }}\text{ }\text{=}\text{ }\text{A}_{\text{MD,0}}\text{n}^{\text{3}}\frac{\text{I}_{\text{tot}}}{\text{I}_{\text{MD}}}$ (1)

$\text{k}_{\text{nr}}\text{ = }\frac{\text{1}}{\text{t}_{\text{obs}}}-\text{ }\text{k}_{\text{r}}$ (2)

$\text{F}_{\text{f-f}}\text{ = }\frac{\text{k}_{\text{r}}}{\text{k}_{\text{r}}\text{ }\text{+}\text{ }\text{k}_{\text{nr}}}$ (3)

where *n* is the refractive index of the medium (*n* = 1.5), *A*_MD,0_ is the spontaneous luminescence probability for the ^5^D_0_→^7^F_1_ transition *in vacuo* (14.65 s^−1^), and (*I*_tot_*/I*_MD_*)* is the ratio of the total area of the Eu^3+^ luminescence spectrum to the area of the ^5^D_0_→^7^F_1_ transition band.^1^

**Supplementary Table S1.** Summary of photophysical parameters of a Eu^3+^-based WMC on a polyolefin-type plastic covering film.

| Samples | τ_obs_ / ms | *k*_r_ / s^−1^ | *k*_nr_ / s^−1^ | Φ*_f_*_-_*_f_* / % |
| --- | --- | --- | --- | --- |
| Eu^3+^-based WCM film | 0.81 | 7.9×10^2^ | 4.4×10^2^ | 64 |
| Eu^3+^-based WCM film*^a^* | 0.58  (0.13 (20%), 0.69 (80%)) | 1.1×10^3^ | 6.3×10^2^ | 64 |
| [**Eu(hfa)_3_(TPPO)_2_**]*^b^* | 0.72 | 1.0×10^3^ | 3.6×10^2^ | 74 |

*^a^*The sample was measured after Japanese larch tree growth experiment.

*^b^*The sample was measured in the solid state without further purification of commercially available [**Eu(hfa)_3_(TPPO)_2_**].

**Supplementary Table S2.** Summary of emission quantum yields of a Eu^3+^-based WMC on a polyolefin-type plastic covering film (λ_ex_ = 330 nm).

| Samples | Φ_tot_ / % |
| --- | --- |
| Eu^3+^-based WCM film | 26 |
| Eu^3+^-based WCM film*^a^* | 1.0 |

*^a^*The sample was measured after Japanese larch tree growth experiment.


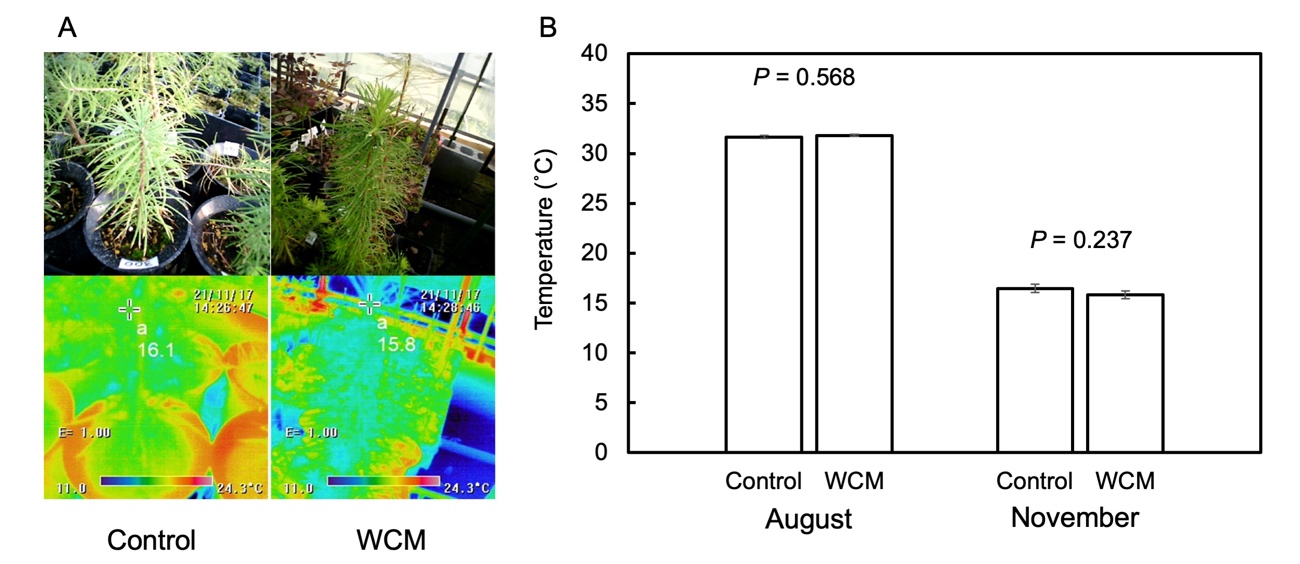


**Supplementary Figure S9.** Temperature of needle-leaves surface on larch seedlings under sunlit of two middays, 7 August and 17 November 2021. Photographs show an example of thermography of larch seedlings covered by plastic films without WCM (control) and with WCM in November (Panel A). In panel B, average temperature of 13–15 seedlings per treatment was shown with standard error. The statistic analysis was examined by generalized linear mixed model.

**Reference**

1. Werts, M. H. V., Jukes, R. T. F., Verhoeven, J. W. The emission spectrum and the radiative lifetime of Eu^3+^ in luminescent lanthanide complexes, *Phys. Chem. Chem. Phys.* **4**, 1542–1548 (2002).
